# Effects of Silica-Particle Coating on a Silica Support for the Fabrication of High-Performance Silicalite-1 Membranes by Gel-Free Steam-Assisted Conversion

**DOI:** 10.3390/membranes9040046

**Published:** 2019-04-01

**Authors:** Kyohei Ueno, Hideyuki Negishi, Takuya Okuno, Hiromasa Tawarayama, Shinji Ishikawa, Manabu Miyamoto, Shigeyuki Uemiya, Yasunori Oumi

**Affiliations:** 1 Department of Oral Biochemistry, Division of Oral Structure, Function and Development, Asahi University School of Dentistry, 1851 Hozumi, Mizuho, Gifu 501-0296, Japan; ueno@dent.asahi-u.ac.jp; 2 Faculty of Engineering, Gifu University, 1-1 Yanagido, Gifu 501-1193, Japan; m_miya@gifu-u.ac.jp (M.M.); uemiya@gifu-u.ac.jp (S.U.); 3 Research Institute for Chemical Process Technology, National Institute of Advanced Industrial Science and Technology (AIST), AIST Central 5, 1-1-1 Higashi, Tsukuba, Ibaraki 305-8565, Japan; h-negishi@aist.go.jp; 4 Frontier Technologies Laboratory, Sumitomo Electric Industries, Ltd., 1, Taya-cho, Sakae-ku, Yokohama, Kanagawa 244-8588, Japan; okuno-takuya@sei.co.jp (T.O.); tawarayama-hiromasa@sei.co.jp (H.T.); ishishin@sei.co.jp (S.I.); 5 Organization for Research and Community Development, 1-1 Yanagido, Gifu 501-1193, Japan

**Keywords:** MFI-type zeolite, silicalite-1 membrane, electrophoretic deposition, tubular silica support, silica particles, gel-free synthesis, steam-assisted conversion, secondary growth, ethanol/water separation, pervaporation

## Abstract

Silicalite-1 membranes with high pervaporation performance were prepared successfully on a silica-particle-coated tubular silica support using a gel-free steam-assisted conversion (SAC) method. The effects of the silica-particle layer formed on the top surface of the silica support and the physical properties of the silica particles themselves on the membrane-formation process were investigated. The silica particles coated served as the additional silica source for growing the silicalite-1 seed crystal layer into the silicalite-1 membrane. As a result, it was possible to form a dense and continuous membrane even under gel-free conditions. Furthermore, it was found that the properties of the silica particles, such as their primary particle diameter, had a determining effect on their solubility during the steam treatment, that is, on the supply rate of the silica source. The silicalite-1 membrane obtained using the spherical-silica-particle-coated support had an approximately 9-μm-thick separation layer and showed very high pervaporation performance, exhibiting a separation factor of 105 and a flux of 3.72 kg m^−2^ h^−1^ for a 10 wt % ethanol/water mixture at 323 K. Thus, the gel-free SAC method can be used with a silica support coated with silica particles to readily prepare high-performance membranes without producing any chemical waste.

## 1. Introduction

Zeolites, which are crystalline aluminosilicates, have unique physical and chemical properties, exhibit a molecular sieving effect, and show hydrophilicity, hydrophobicity, and solid acidity owing to the presence of uniform, molecular-sized pores and Al in the zeolite structure. Thus, they are used widely as adsorbents, catalysts, and membrane materials. In particular, they are highly suitable for producing membranes that show higher thermal, mechanical, and chemical stabilities than polymeric membranes [1,2]. MFI-type zeolite is an industrially important zeolite whose hydrophilicity and hydrophobicity can be controlled readily by varying the amount of Al present in its structure. In particular, siliceous MFI-type zeolite (called silicalite-1) shows high hydrophobicity and selectively adsorbs nonpolar molecules such as organic molecules. Therefore, silicalite-1 membranes are employed widely for separating organic molecules from organic/water mixtures [3,4,5,6,7,8,9,10,11,12,13,14,15,16,17,18,19,20,21,22,23,24,25], especially ethanol/water mixtures.

Silicalite-1 membranes are generally prepared by hydrothermal synthesis (HT) using an in-situ crystallization or secondary growth method [3,4,5,6,7,8,9,10,11,12,13,14,15,16,17,18,19,20,21,22,23,24,25]. In both cases, the synthetic gel is prepared by mixing a silica source, a structure-directing agent (SDA), a mineralizing agent, and water and immersing the support in this mixture to synthesize the membrane. After crystallization, the membrane is washed with a large amount of water to remove the synthetic gel. Most of the silica source is used for the synthesis of unwanted silicalite-1 powder, and the remaining highly basic synthetic gel and the washings need to be neutralized with an acid and subsequently discarded. Thus, although HT is an established method for the synthesis of zeolite membranes, it involves the use and subsequent treatment of large amounts of the synthetic gel, which is an alkali-mixed solution containing an expensive SDA. This and the relatively low utilization efficiency of the initial synthetic gel are the primary factors limiting the applicability of zeolite membranes.

Recently, a gel-free approach was developed [26,27,28] that is gradually attracting a great deal of interest, as it is very simple, does not waste chemicals, and readily allows for the scaling-up of production [26]. However, this gel-free method can only be used with flat-plate-type supports, and it is necessary to modify it for tubular supports, which have larger surface areas and are thus better suited for industrial production. More recently, our group succeeded in developing a novel membrane synthesis procedure under gel-free conditions called the gel-free steam-assisted conversion (SAC) method, which uses a novel tubular silica support [22]. Specifically, in this method, a silicalite-1 membrane is formed through a steam treatment while using a silicalite-1-seeded silica support coated with an aqueous tetra-n-propylammoniumhydroxid (TPAOH) solution. In this method, the silica support is not only used as the support but also as the silica source to grow the seed layer into a continuous membrane. That is to say, the surface of the silica support coated with TPAOH dissolves and is used up as the silica source. Membranes synthesized by this process showed high flux (4.47 kg m^−2^ h^−1^) during the separation of an ethanol/water mixture; however, the separation factor (α = 66) was slightly lower than that of membranes prepared by the HT technique [5,6,7,8,14,15,16,17]. In the case of membrane synthesis by the HT method, since the seeded support is immersed in a synthetic gel containing the silica source in a sufficient amount, the membrane layer generally grows with an increase in the synthesis time, owing to which the separation factor is higher. However, in the gel-free SAC method, because the silica source is supplied from the top surface of the silica support and is thus limited, for synthesis times greater than a certain threshold, the separation factor does not improve [22]. From this result, it can be concluded that, when using this fabrication method, it is necessary to increase the amount of silica source supplied for the growth of the seed crystal in order to improve the separation performance. To the best of our knowledge, there is no other report of the gel-free synthesis of such membranes on a tubular support, and determining the optimal conditions for improving the separation performance of these membranes would contribute greatly to the development of this method.

Here, in order to further expand the applicability of the gel-free SAC method, we synthesized silicalite-1 membranes using a tubular silica support coated with silica particles. The silica particles coated on the support acted as the additional silica source for growing the seed crystals into a membrane. Furthermore, the coating of the silica particles on the top surface of the support not only increased the supply of the silica source by increasing the number density of silica particles on the support surface, but also resulted in the synthesis of a denser, more continuous membrane by smoothening the top surface of the support. The effects of the silica-particle layer as well as those of the properties of the silica particles themselves on membrane growth and the separation performance of the resulting membranes were also investigated in this study. Further, the separation performance of the synthesized membranes was evaluated with respect to the separation of an ethanol/water mixture by pervaporation (PV).

## 2. Materials and Methods 

### 2.1. Formation of Silica-Particle Coating on Silica Support

Two types of silica particles (spherical silica, SAKAI CHEMICAL INDUSTRY CO., LTD., Osaka, Japan and fumed silica, Nippon Aerosil Co., Ltd., Tokyo, Japan) were used in this study to coat the top surface of the silica support; their characteristics are listed in Table 1. The fumed silica particles had a primary particle diameter of approximately 7 nm; however, these agglomerated to form larger secondary particles. The coating of the silica particles on the top surface of the tubular silica support (length: 80 mm, outer diameter: 10 mm, inner diameter: 8.4 mm, porosity: 64%, pore size: 0.5 μm; Sumitomo Electric Ind., Japan) was formed as per the following procedure. First, a silica-particle dispersion (1.2 wt %) was prepared by dispersing the particles in question in distilled water by ultrasonication for 5 min. The resulting dispersion was poured over a piece of nonwoven fabric and applied on the outer surface of the support by rubbing. The silica-particle-coated support was then dried at room temperature for 30 min and subsequently calcined at 823 K for 2 h to increase the bonding strength between the support and the coated silica particles. Thus, it can be seen that the procedure used in this study to prepare the support is a very simple one.

### 2.2. Preparation of Seeded Silica-Particle-Coated Support by Electrophoretic Deposition

Seed crystals of silicalite-1 (average particle diameter of 1.0 μm) were prepared according to a previously described procedure [15]. The deposition of the seed crystals on the silica-particle-coated tubular silica support was performed by electrophoretic deposition (EPD) [15]. Prior to the EPD process, a zeolite-containing acetone solvent with a concentration of 5 g L^−1^ was prepared by ultrasonic dispersion for 30 min. During the EPD process, a direct current voltage of 50 V was applied for 3 min in order to deposit the seed crystals on the outer surface of the support. The optimal seed loading amount per unit outer surface area of the silica support for preparing silicalite-1 membranes on silica supports by the gel-free SAC method was previously reported to be approximately 2 g m^−2^ [22]. Therefore, in this study too, the loading amount of the seed crystals on the silica-particle-coated silica support was adjusted to 2 g m^−2^. The obtained silicalite-1-seeded silica-particle-coated silica support was calcined at 573 K for 6 h to increase the adhesion strength between the silica-particle-coated support and the silicalite-1 seed crystals.

### 2.3. Synthesis of Silicalite-1 Membrane by Gel-Free SAC Method

**Figure 1:**
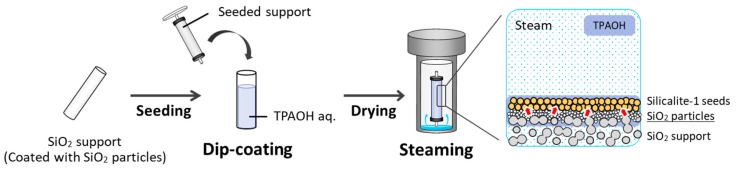
Schematic of process for synthesizing silicalite-1 membranes using the gel-free SAC method.

The synthesis of the silicalite-1 membranes by the gel-free SAC method was performed as follows (Figure 1). The seeded silica support was first coated by dip coating in a 0.1 M aqueous TPAOH solution for 30 s. Next, the TPAOH-coated seeded support was dried for 1 h at 333 K and then placed in an autoclave containing 3 g of water at the bottom. Subsequently, crystallization was performed at 433 K for 16 or 24 h. In this method, both the silica support and the silica particles coated on its surface acted as silica sources, while TPAOH acted as both a SDA and the base. Further, the necessary water was supplied by the formed water vapor. After the crystallization process was complete, the synthesized membrane was removed from the autoclave, washed with a small amount of water, dried overnight at 333 K, and then calcined at 648 K for 40 h to remove the SDA from the zeolite pores.

### 2.4. Membrane Characterization

The synthesized membranes were characterized, that is, their zeolite phases and morphologies were identified using X-ray diffraction (XRD) analysis (D8 Advance, Bruker AXS, Karlsruhe, Germany) and scanning electron microscopy (SEM) imaging (S-4800, Hitachi High-Technologies, Tokyo, Japan), respectively. The separation performances of the membranes were evaluated based on the PV separation of an ethanol/water mixture (10/90 wt %) at 323 K. The permeated vapor was collected using a liquid-nitrogen-cooled cold trap. The ethanol concentrations of the feeds and permeates were calculated by liquid chromatography (Shimadzu, Tokyo, Japan). The flux (J) is defined as follows:

J = m/(A·t)(1)

where m is the total amount of the permeate (kg), A is the effective membrane area (m^2^), and t is the operating time (h). The PV separation factor (α) is defined as follows:

α = (Y_ethanol_/Y_water_)/(X_ethanol_/X_water_) (2)

where X and Y are the weight fractions of the species in the feed and permeate, respectively. The PV separation factor (α) as defined above can be divided further into two terms, namely, the evaporation separation factor (α_evap_) and the intrinsic membrane selectivity (α_mem_); the evaporation separation factor (α_evap_) is a function of the feed conditions and can be determined from the vapor–liquid equilibrium data [28].

α = α_evap_ × α_mem_ (3)

## 3. Results and Discussion

### 3.1. Silica-Particle-Coated Support

**Figure 2:**
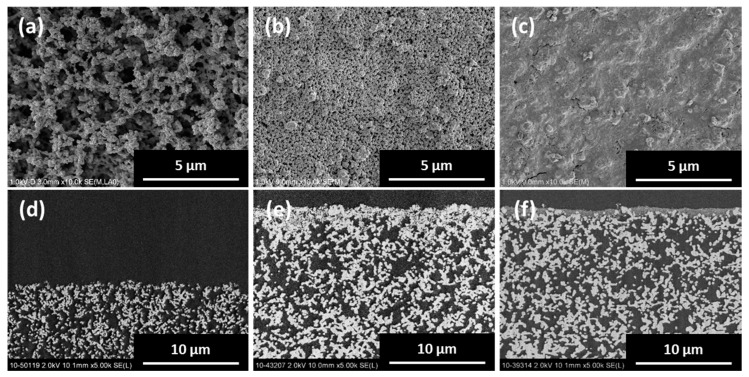
Top (upper row) and cross-sectional (lower row) SEM images of silica support (**a**,**d**) before and after coating of top surface with (**b**,**e**) spherical silica and (**c**,**f**) fumed silica.

Figure 2 shows SEM images of the silica support before and after the formation of a coating of silica particles on its top surface. In order to elucidate the deposition state of the silica particles on the silica support, a silica-particle-coated support was embedded with a resin. It was then subjected to cutting and surface polishing and subsequently to SEM imaging (Figure 2d–f; the white, gray, and black contrasts correspond to the silica support, coated silica, and pores, respectively). It can be seen that the silica support has an open-pore structure, which is formed by the sintering of the submicron-sized silica particles (Figure 2a). After the covering of the silica support surface with the silica particles, regardless of the size of the particles used, a silica-particle layer was formed on the top surface, with the thickness of this layer being approximately 2 μm (Figure 2e,f). Furthermore, after the formation of this layer, the top surface of the silica support became smoother, and the number density of the silica particles increased (Figure 2b,c). The size of the primary particles of the fumed silica sample was approximately 7 nm; however, these particles did not penetrate the pores of the silica support and could only coat its top surface. This is because, while the primary particles were small, they agglomerated to form larger secondary particles. From these results, it can be concluded that the formation of a coating of silica particles on the silica support not only increases the number density of the silica particles on the support surface but also results in a smoother support surface, which is advantageous for the subsequent formation of a continuous and dense seed layer. This, in turn, results in the formation of a continuous zeolite membrane. The silica supports coated with silica particles were used for the subsequent experiments.

### 3.2. Silicalite-1 Membrane

**Figure 3:**
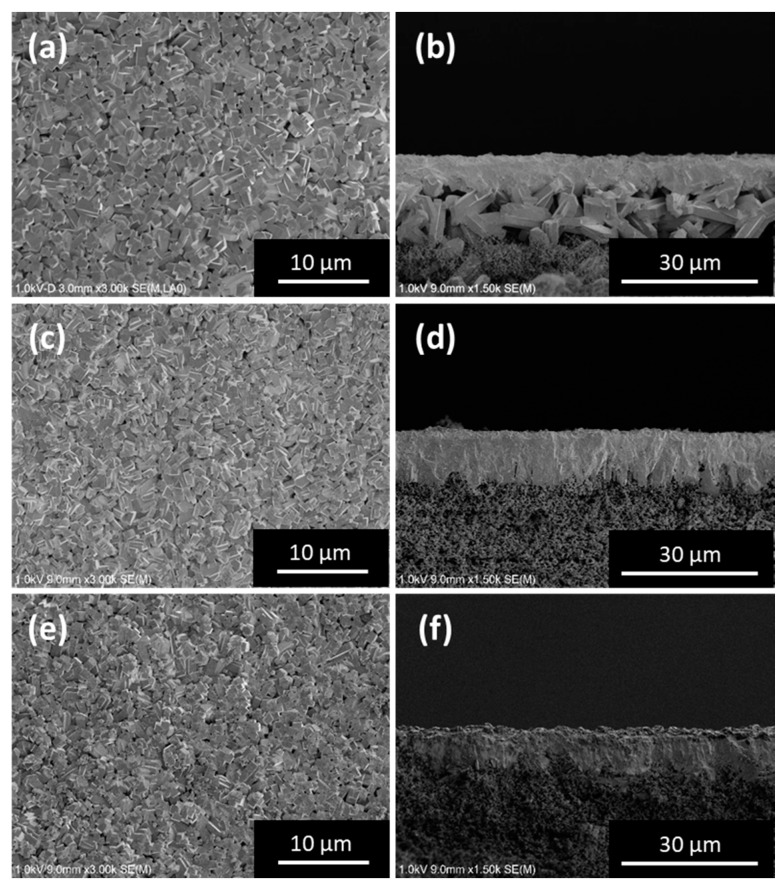
Top (left) and cross-sectional (right) SEM images of silicalite-1 membranes fabricated using uncoated and silica-particle-coated (different types of particles) silica supports by gel-free SAC method. (**a**,**b**) Uncoated silica support for 24 h, (**c**,**d**) spherical-silica-particle-coated silica support for 24 h, and (**e**,**f**) fumed-silica-particle-coated silica support for 16 h.

**Figure 4:**
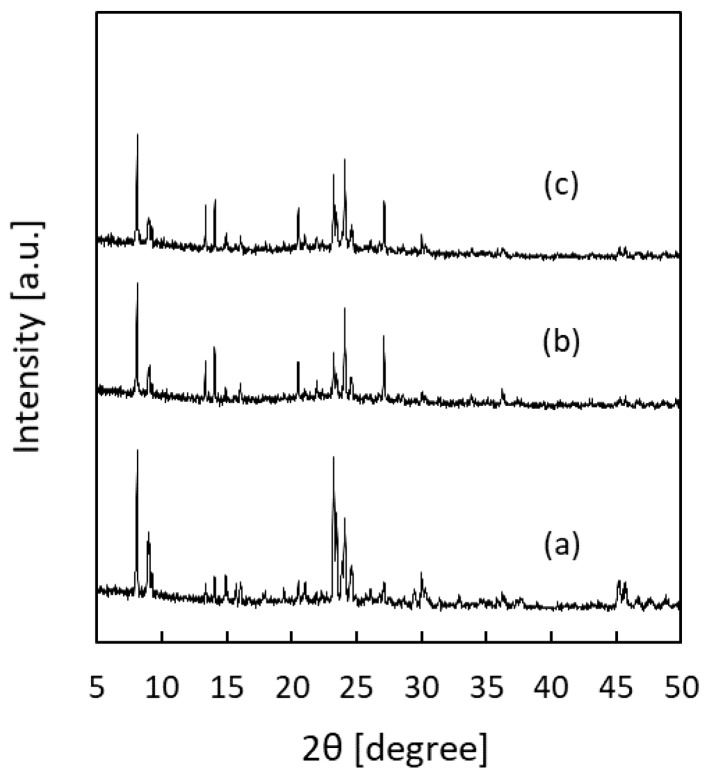
XRD patterns of silicalite-1 membranes prepared by the gel-free SAC method using different silica supports. (**a**) Uncoated support for 24 h, (**b**) spherical-silica-particle-coated support for 24 h, and (**c**) fumed-silica-particle-coated support for 16 h.

Figure 3 and Figure 4 show SEM images and XRD patterns, respectively, of the silicalite-1 membranes prepared using the uncoated and different silica-particle-coated silica supports under different synthesis conditions (16 or 24 h at 433 K). Based on the top-surface SEM images of the supports taken after secondary growth (Figure 3a,c,e), it can be seen that the seed crystals on the support underwent growth and that the membrane surface was covered with the intergrown crystals. The surface morphologies of the membranes grown using supports with and without a silica-particle coating were not significantly different. However, their cross-sectional morphologies were different. In the case where an uncoated support was used (synthesis time: 24 h), the formation of columnar zeolite crystals characteristic of silicalite-1 was observed between the continuous silicalite-1 membrane layer and the silica support, giving the membrane a specific hierarchical structure (Figure 3b and Figure S1). To elucidate the formation process of these columnar crystals on the support, we attempted to use the gel-free SAC method on an unseeded and uncoated silica support while varying the synthesis time. The XRD patterns (Figure S2) of the obtained product contained peaks typical of an MFI-type zeolite structure, with no other phases being present. Further, the crystallinity increased with the synthesis time. Moreover, SEM images (Figure S3) showed that these columnar crystals formed on the top surface of the support as the synthesis time was increased. Therefore, it can be concluded that this layer of columnar crystals was formed because of the conversion of the silica support itself into MFI-type zeolite crystals by the steam treatment. If the formation of this layer of columnar crystals were to be promoted, the bonding strength between the continuous membrane layer and the support would weaken, resulting in the peeling of the membrane layer from the support. On the other hand, in the case of the use of a silica support coated with silica particles, the fabricated membranes consisted only of a continuous membrane layer (Figure 3d,f). That is to say, columnar zeolite crystals derived from the silica support were not formed when a silica support coated with silica particles was used. In addition, no silica particles were present on the top surface of the silica support after the steaming treatment. This suggested that all the coated silica particles had been consumed during the growth of the silicalite-1 membrane, confirming that the coated silica particles had acted as the silica source for the growth of the seed crystal layer and the subsequent formation of the membrane during the steaming process. Furthermore, the consumption of the coated silica particles as well as the top surface of the silica support itself resulted in direct and hence strong bonding between the bottom of the fabricated silicalite-1 membrane layer and the top of the silica support.

The XRD patterns of the membranes fabricated using the uncoated and coated silica supports contained peaks typical of the MFI-type zeolite structure. Further, no peaks related to other phases were present, and this was the case regardless whether a coating had been used or not (Figure 4). However, the orientation of the membranes prepared on the coated silica supports (Figure 4b,c) was significantly different from that of the membrane prepared on an uncoated support (Figure 4a). Based on the SEM images (Figure 3b,d,f), this difference in the orientations can be attributed to the existence of columnar MFI-type zeolite crystals between the continuous zeolite membrane layer and the support in the case of the uncoated support.

Compared to the case for the uncoated support, when the spherical-silica-particle-coated support was used, the thickness of the membrane layer increased from 7 μm (uncoated silica support) to 9 μm. Further, there was no crystallization of the support itself, even for a synthesis time of 24 h (Figure 3d). From these results, it may be concluded that the coating of the silica particles formed on the top surface of the support dissolves during the steaming treatment, acting as an additional silica source. Moreover, TPAOH was adsorbed preferentially onto the silica particles coated on the support, thereby preventing the excessive dissolution and crystallization of the underlying silica support. In other words, this phenomenon limits the lowering of the mechanical strength of the silicalite-1 membranes prepared using the gel-free SAC method. In addition, when the fumed silica particles, which had a smaller primary particle size, were used, a membrane similar to that formed using the spherical-silica-particle-coated support after 24 h could be produced in a shorter synthesis time (16 h). Since the fumed silica particles were smaller and thus had a larger specific surface area, they probably dissolved more readily during the steaming treatment, owing to which the membrane-formation reaction reached completion in a shorter time. As stated above, it can thus be concluded that the physicochemical characteristics of the silica particles used for coating the silica support have a determining effect on the membrane-formation process.

### 3.3. Pervaporation Performance

The PV performances of the silicalite-1 membranes prepared on the silica-particle-coated silica supports were evaluated using a 10 wt % ethanol feed solution at 323 K (Table 2). The membrane (M1) prepared using the spherical-silica-particle-coated support for 24 h showed excellent PV performance, exhibiting a separation factor of 105 and a flux of 3.72 kg m^−2^ h^−1^. Further, its separation factor was markedly higher than that of the membrane (M3) formed on the uncoated silica support. This result also suggests that the silica particles coated on the silica support effectively functioned as a silica source for the growth of the seed crystal layer into the membrane layer during the steaming treatment and contributed significantly to the formation of a higher-quality silicalite-1 membrane. In addition, even when the fumed silica particles with a smaller size were used as the coating, the separation factor of the resulting membrane (M2) was significantly better than that of the membrane (M4) formed on the uncoated support under similar conditions. Further, it was found that it is possible to fabricate a membrane with a PV performance equivalent to that of the membrane prepared using spherical silica particles in only 16 h. From these results, it is clear that the physicochemical properties of the silica particles used for coating the silica support have a significant effect on the solubility of the silica particles during the steaming treatment and hence the supply rate of silica, which is a critical factor for preparing high-performance silicalite-1 membranes. Moreover, regardless of the type of silica particles used, an increase in the synthesis time did not result in any improvements in the PV performance of the membranes. This is probably because, once all the raw materials for growing the seed crystal layer into the membrane layer had been consumed, the steaming treatment only served to reduce the membrane properties, as reported in our previous work [22].

**Figure 5:**
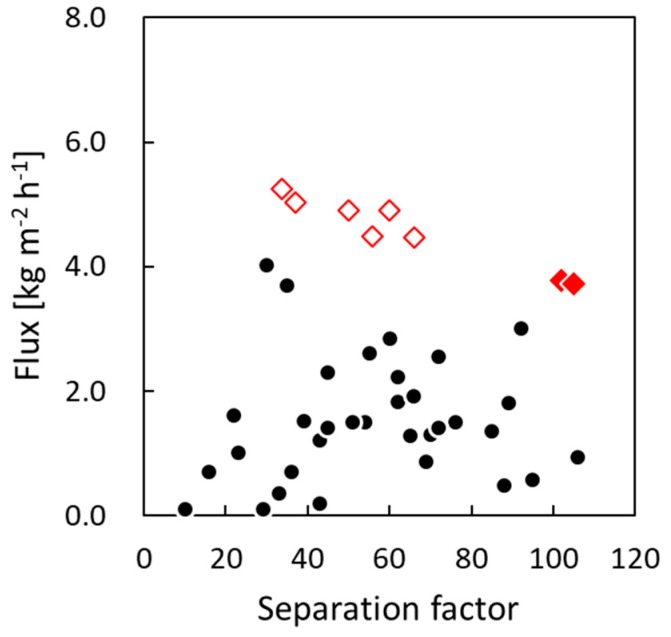
Relationship between the separation factor (α) and the flux through silicalite-1 membranes during separation of ethanol/water mixtures (●: hydrothermal synthesis [3,4,5,6,7,8,9,10,11,12,13,14,15,16,17,18,19,20,21]; ◇: gel-free SAC (uncoated silica support) [22]; ◆: gel-free SAC (silica-particle-coated silica support)).

The PV performances of the membranes fabricated in the present study were also compared with those of silicalite-1 membranes prepared on tubular supports by HT [3,4,5,6,7,8,9,10,11,12,13,14,15,16,17,18,19,20,21] and on silica supports by the gel-free SAC method [22] in previous studies (Figure 5). The silicalite-1 membranes fabricated in this study on the silica-particle-coated supports using the gel-free SAC method showed very high PV performances as well as highest membrane selectivity (α_mem_) compared with the previously reported membranes [28]. This was because, in the present study, the high-quality silicalite-1 membrane layer, which was dense and continuous, was composed of pure silica. Moreover, in general, there is a trade-off relationship between the separation factor of membranes and their flux during the separation of ethanol/water mixtures [16,29]. That is to say, the separation factor increases and the flux decreases with an improvement in the quality of the membrane layer. As can be seen from Figure 5, there did exist a trade-off relationship between the separation factor and the flux of the silicalite-1 membranes prepared on silica supports (uncoated and silica-particle-coated) using the gel-free SAC method. There was no significant reduction in the flux of the membranes prepared on the silica-coated support as compared to the PV performance estimated from the performance of the membranes prepared on the uncoated silica support. Thus, the permeability of the silica support itself did not decrease. From this result and the SEM images in Figure 3d,f, it can be concluded that, in the present study, almost all silica particles coated on the support were consumed during the membrane-formation process. 

### 3.4. Cost of Synthesis of Silicalite-1 Membranes

**Figure 6:**
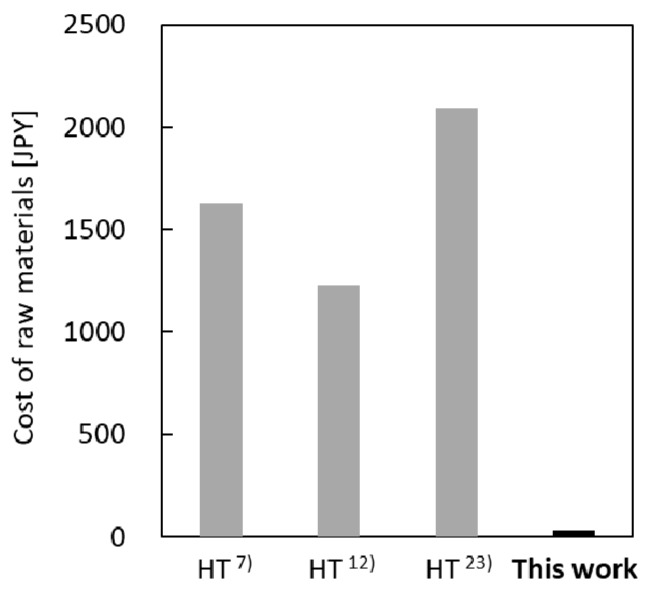
Comparison of raw-material costs for preparation of silicalite-1 membranes (hydrothermal synthesis (HT): gray bar; gel-free SAC: black bar).

For zeolite membranes to be practical for industrial applications, their cost of preparation must be low. In the conventional HT method, typically, a synthetic gel is prepared by mixing tetraethyl orthosilicate as the silica source, TPAOH as the SDA, an alkali source, and water [7,12,23]. On the other hand, when using the gel-free SAC method, there is no need for a silica source derived from a synthetic gel, and it is possible to prepare membranes using a small amount of water and the minimum necessary amount of TPAOH. Assuming that a reaction vessel of the same size was used, the raw-material cost (excluding that of the support) per membrane for the membranes fabricated by the gel-free SAC and HT methods were compared (Figure 6). The raw-material costs were calculated based on the prices quoted by SIGMA-ALDRICH, Japan. It was found that the raw-material cost per membrane in the case of the gel-free SAC method is much lower than that for the HT method. Furthermore, in the case of the HT method, after membrane synthesis, it is necessary to wash the membrane with a large amount of water. In the gel-free SAC method, however, it is sufficient to wash the membrane with a small amount of water, and it is not necessary to wash the reaction vessel with an alkaline solution, as only water remains in the vessel. The gel-free SAC method, which is superior in terms of simplicity and cost, is thus more useful than the conventional HT method for fabricating silicalite-1 membranes. Moreover, for improving the PV performance of silicalite-1 membranes prepared using the gel-free SAC method, the coating of silica particles on the silica support is highly effective. However, further studies, including those on the optimization of the thickness of the silica-particle layer, the type of silica particles to be used, and the amount of TPAOH adsorbed onto the silica particles are necessary to improve the industrial applicability of these membranes.

## 4. Conclusions

Silicalite-1 membranes with high PV performances were prepared on silica-particle-coated tubular silica supports using a gel-free SAC method. In this study, the effects of the silica-particle layer formed on the top surface of the silica support and the physical properties of the silica particles themselves on the membrane formation process were investigated. The silica particles coated on the silica support functioned as the additional silica source for growing the silicalite-1 seed crystal layer into the silicalite-1 membrane. As a result, it was possible to form dense and continuous membranes even under gel-free conditions. Furthermore, it was found that the properties of the silica particles coated on the top surface of the silica support determine their solubility during the steam treatment and hence the supply rate of the silica source. When fumed silica particles having a smaller primary particle size and a larger specific surface area were used to coat the top surface of the silica support, high-performance membranes could be synthesized in a shorter time. The silicalite-1 membranes obtained using the spherical-silica-particle-coated support fabricated in this study had a separation layer with a thickness of approximately 9 μm and exhibited very high PV performance, having a separation factor of 105 and a flux of 3.72 kg m^−2^ h^−1^ for a 10 wt % ethanol/water mixture at 323 K. Thus, the gel-free SAC method can be used with a silica support coated with silica particles to readily prepare high-performance membranes without producing any chemical waste. Therefore, we believe that the proposed method will aid the development of an economical and environmentally friendly method for fabricating zeolite membranes on an industrial scale.

## Figures and Tables

**Table 1:** Characteristics of silica particles used as coating materials in this study.

Coating Material	Average Primary Particle Diameter [nm]	BET Specific Surface Area [g m^−2^]
Spherical silica	100	22
Fumed silica	7	380

**Table 2:** PV performances of silicalite-1 membranes prepared under different synthesis conditions using the gel-free SAC method with respect to separation of ethanol/water mixture.

Sample Name	Coating Material	Synthesis Time [h]	Separation Factor (α)	Membrane Selectivity (α_mem_)	Flux [kg m^−2^ h^−1^]	Ref.
M1	Spherical silica	24	105	10.6	3.72	This study
M2	Fumed silica	16	102	10.3	3.76	This study
M3	–	24	66	6.6	4.46	[22]
M4	–	16	56	5.6	4.49	[22]

## Supplementary Materials

The following are available online at https://www.mdpi.com/2077-0375/9/4/46/s1. Figure S1: SEM image of silicalite-1 membrane fabricated by the gel-free SAC method using uncoated silica support; Figure S2: XRD patterns of obtained products prepared by the gel-free SAC method for different synthesis times using unseeded uncoated silica support; Figure S3: SEM images of obtained products prepared by the gel-free SAC method for different synthesis times using unseeded uncoated silica support.
