# Supplementary material for: Effects of Silica-Particle Coating on a Silica Support for the Fabrication of High-Performance Silicalite-1 Membranes by Gel-Free Steam-Assisted Conversion
Source: Membranes (Basel). 2019 Apr 1;9(4):46. doi: 10.3390/membranes9040046 (PMC6523581; doi:10.3390/membranes9040046)
Supplement: Supplementary file 1 [file membranes-09-00046-s001.pdf]

Supplementary material

Effects of Silica-Particle Coating on a Silica Support for the Fabrication of High-Performance Silicalite-1 Membranes by Gel-Free Steam-Assisted Conversion

Kyohei Ueno [1,2], Hideyuki Negishi [3], Takuya Okuno [4], Hiromasa Tawarayama [4], Shinji Ishikawa [4], Manabu Miyamoto [2], Shigeyuki Uemiya [2] and Yasunori Oumi [5,*]

1 Department of Oral Biochemistry, Division of Oral Structure, Function and Development, Asahi University School of Dentistry, 1851 Hozumi, Mizuho, Gifu 501-0296, Japan; ueno@dent.asahi-u.ac.jp
2 Faculty of Engineering, Gifu University, 1-1 Yanagido, Gifu 501-1193, Japan; m_miya@gifu-u.ac.jp (M.M.); uemiya@gifu-u.ac.jp (S.U.)
3 Research Institute for Chemical Process Technology, National Institute of Advanced Industrial Science and Technology (AIST), AIST Central 5, 1-1-1 Higashi, Tsukuba, Ibaraki 305-8565, Japan; h-negishi@aist.go.jp
4 Frontier Technologies Laboratory, Sumitomo Electric Industries, Ltd., 1, Taya-cho, Sakae-ku, Yokohama, Kanagawa 244-8588, Japan; okuno-takuya@sei.co.jp (T.O.); tawarayama-hiromasa@sei.co.jp (H.T.); ishishin@sei.co.jp (S.I.)
5 Organization for Research and Community Development, 1-1 Yanagido, Gifu 501-1193, Japan
* Correspondence: oumi@gifu-u.ac.jp; Tel.: +81-58-293-3335

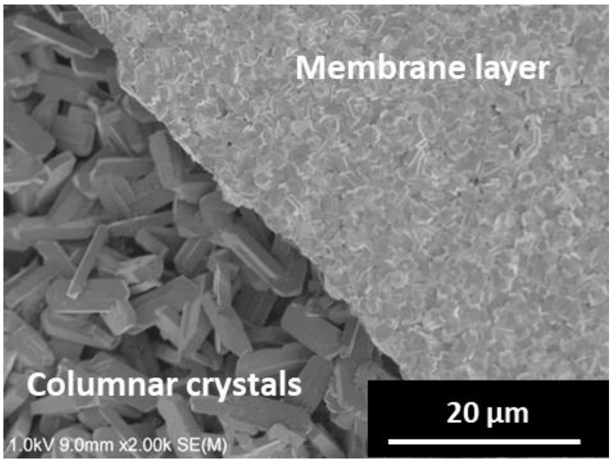

Figure S1. SEM image of silicalite-1 membrane fabricated by gel-free SAC method using uncoated silica support.

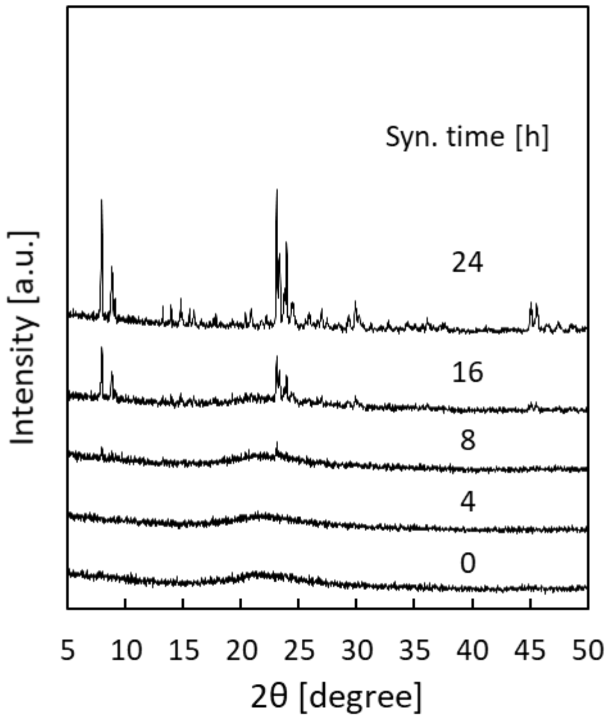

Figure S2. XRD patterns of obtained products prepared by gel-free SAC method for different synthesis times using unseeded uncoated silica support.

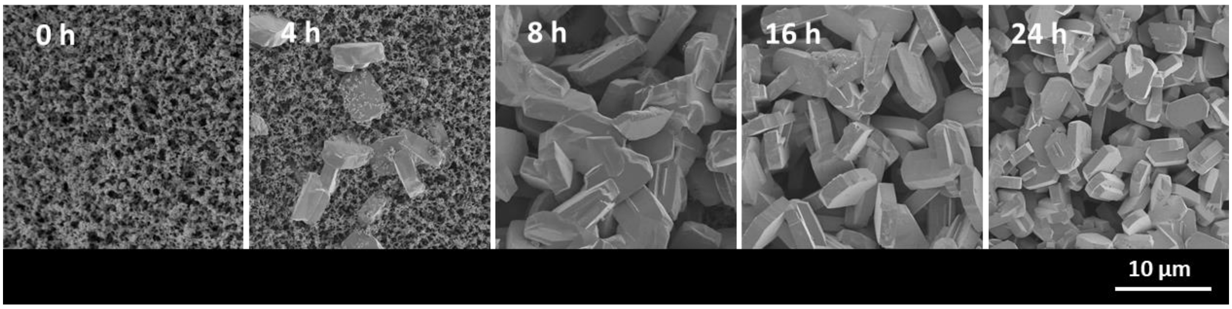

Figure S3. SEM images of obtained products prepared by gel-free SAC method for different synthesis times using unseeded uncoated silica support.
